# Facile One Pot Greener Synthesis of Sophorolipid Capped Gold Nanoparticles and its Antimicrobial Activity having Special Efficacy Against Gram Negative *Vibrio cholerae*

**DOI:** 10.1038/s41598-019-57399-3

**Published:** 2020-01-29

**Authors:** Sristy Shikha, Saumya Ray Chaudhuri, Mani Shankar Bhattacharyya

**Affiliations:** CSIR-Institute of Microbial Technology (IMTECH), Sector-39A, Chandigarh, 160036 India

**Keywords:** Nanoparticles, Antibiotics

## Abstract

Microbes develop several strategies to survive in the adverse condition such as biofilm formation, attaining non-dividing state, altering drug target or drug, thereby increases the burden of drug dosage. To combat these issues, nanoparticles have shown an alternative approach for new treatment strategy but synthesis via chemical synthetic route limits their application in biomedical field. Here, green method for the synthesis of gold nanoparticles using sophorolipid (SL) is discussed that is characterized by various techniques. Initially, the antimicrobial activity was checked against metabolically active state of microbes; Gram-positive *Staphylococcus aureus* and Gram-negative *Vibrio cholerae* using XTT assay and growth kinetics assay. Results suggested higher efficacy of nanoparticles for Gram-negative, therefore further analyzed against *Escherichia coli* that confirmed its potency for the same. AuNPs-SL also signifies its efficiency at least metabolically active state; non dividing cells and biofilm of these microbes. Induced morphological changes were studied by SEM that revealed AuNPs-SL led to disruption of cell membrane and leakage of intracellular fluid to the surroundings. Inhibition of respiratory enzymes activity also plays a crucial role in bactericidal action as indicated by LDH assay. Synergy of AuNPs-SL with different antibiotics was also analyzed using checkerboard assay. These results suggested the possible use of AuNPs-SL as an antimicrobial therapy in the field of nanomedicine.

## Introduction

In recent years, the emergence of antimicrobial resistance has been recognized as one of the most compelling problem in the field of biomedical sciences. Development of resistance has been reported even against the newly invented drug candidates. The reasons behind the emergence of bacterial resistance are complicated, but it can be discriminated as, partly due to the selection of bacterial mutants by antibiotics and partly due to reduced dosage intake by patients that lead to reduced levels of antibiotics which in turn, trigger bacterial resistance. Multiplying (logarithmic phase) and non-multiplying (stationary phase, dormant or latent) are two different states of bacterial existence^1^. Among different survival strategies under adverse conditions, formation of biofilm and achieving dormant or non dividing phase are the privilege one^2^. A survey conducted by National Institutes of Health and Centre of Disease control suggested that most of the infection (nearly 65–80%) occurred by biofilm formation^3^. Biofilms are complex mixtures of bacteria, containing both states of bacterial existence; multiplying and non-multiplying bacteria^4^. In biofilm, cellular metabolic activity is reduced and cells are more resistant to drugs. A significant proportion of most human bacterial infectious disease (nearly 60%)^5^ is encompassed by non-multiplying bacteria and these are not easily killed by antibiotics^6^. Multiplying bacteria are killed easily in presence of antibiotics as compared to non-multiplying ones. Therefore, designing better therapeutic strategies, and/or modification and augmentation of the existing therapies are highly essential to combat these problems. As an alternative to the existing therapies, nanoparticles have shown greater potential to combat with the problem and are presently being used for several purposes such as; diagnosis of diseases, cancer sensing and therapy^7^, *in vivo* and *in vitro* cell labelling^8^, antimicrobial agents in implants^9^, cosmetics^10^, food^11^ and fabric industries^12^. Therapeutic efficacy and the mode of action for killing the microbes have given an edge to the nanotherapeutics. Nanoparticles (metallic and non-metallic) are synthesized either chemically or biologically, have shown promising results for antimicrobial activity against diverse spectra of microbes^13,14^. Among these, metal and metal oxide nanoparticles are shown to have a wide range of antimicrobial activity since the ancient time.

The unique characters of metallic nanoparticles like optical, electrical, physio-chemical and ease of surface fabrication with desired molecules, have widen the application window of nanoparticles in several fields such as biosensors^15^, biomedical (as antimicrobials)^14^, electrical^16^, electrochemical energy applications^17^. Recently, gold nanoparticles (AuNPs) have gained profound attention due to high biocompatibility and nontoxic nature. AuNPs are used as carriers of biomolecules (DNA, proteins, peptides and drugs) for cellular imaging, molecular diagnostic, photothermal agents etc^18^. Synthesis of metallic nanoparticles can be achieved mainly by two methods; one is chemical synthesis and the other is green synthesis. Though chemical synthesis is the most frequently used method for nanoparticles synthesis, but involvement of various hazardous chemicals and solvents restricted their application in biomedical fields^19^. The final qualities of the synthesized nanoparticles are determined by the intrinsic nature of metal and capping agents apart from solvent system used for synthesis. Therefore, biological synthesis has emerged as a new strategy for reducing or/and stabilizing the nanoparticles meeting the requirement of clean, least toxic and environmentally benign and thus enhances the horizon of their applications in biomedical fields^20^. The most commonly used ideal alternatives for the greener synthesis of metallic nanoparticles are whole microorganisms (serving as nanofactories) or their products and plant extracts^21,22^. The nature of these extracts is generally starch, protein, lipids or their derivatives. Among Lipid, glycolipids are the most frequently used for greener synthesis of metallic nanoparticles.

Glycolipids are amphiphilic biomolecules having lipids as backbone and carbohydrate as the head part that is linked by a glycosidic bond, in which the carbohydrates moieties can vary from monosaccharide to polysaccharide. One of the important class of glycolipids is sophorolipids (SLs), in which disaccharide sophoroses (2-O-*β*-D-glucopyranosyl-D-glucopyranose) linked *β* glycosidically to the hydroxyl group at the penultimate carbon of fatty acids^23^. Sophorolipids have been well established for biocompatibility and antimicrobial for diverse group of microbes such as bacteria, fungi, viruses^24^. Apart from these, SLs being biodegradable, least ecotoxicity, and cost-effective (as they can be produced using renewable resource substrates) provides an edge over other biomolecules^25^. The US FDA has also approved these biosurfactants/sugar esters for the use in food and pharmaceuticals^26^. Understanding the significance of SLs, it has been used as reducing/capping for the synthesis of different metallic nanoparticles^27^.

From the green chemistry prospect, the fundamental requirements for the preparation of nanoparticles are (i) the choice of solvent medium and (ii) non-toxic reducing and stabilizing agents. In the present work, gold nanoparticles were prepared using sophorolipid as reducing and stabilizing agents that were characterized using different techniques. Two pathogenic strains Gram-positive *Staphylococcus aureus* and Gram-negative *Vibrio cholerae* EL Tor strain N16961 were used as indicator strain for antimicrobial activity. *S. aureus* is well established as human pathogen, known to cause bloodstream infections, pneumonia, or bone and joint infections. *V. cholerae* is the causative agent of cholera; the second most leading cause of mortality among children under five years old and one for the major reasons of morbidity among adults^28^. To further validate the efficacy against Gram negative bacteria of the nanoparticles, *Escherichia coli* was used as another indicator bacterium. Subsequent to this, the potency of nanoparticles on non-dividing cells and biofilm was also studied. SEM and TEM studies were performed to check the morphological changes induced by AuNPs-SL. To understand the mechanism of antibacterial activity, Lactate dehydrogenase assay (LDH) assay was performed. Different drugs having different mechanism of antimicrobial action were used to examine the synergistic effects with AuNPs-SL against these microbes.

## Results

### Synthesis and characterization of sophorolipid capped gold nanoparticles

**Figure 1:**
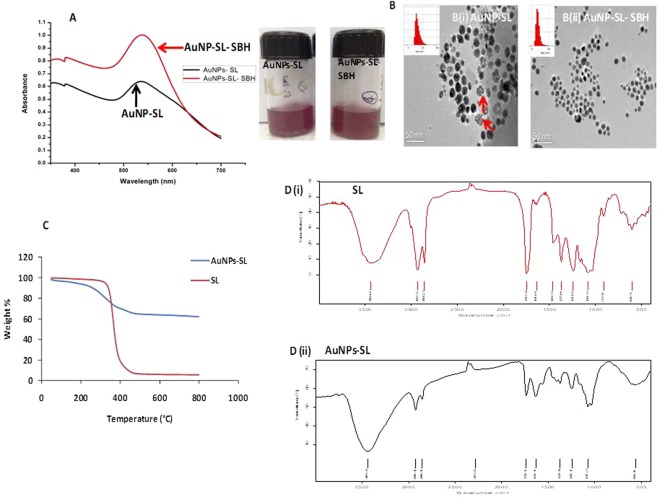
UV-visible spectra of gold nanoparticles synthesized by two different methods as indicated (**A**) TEM images of AuNPs-SL synthesized by two different method in absence (**B**-i) and presence (**B**-ii) of sodium borohydride (indicated as SBH) TGA (**C**) and FTIR spectra of SL (**D**-i) & AuNPs-SL (D-ii).

Synthesis of gold nanoparticles was performed using sophorolipid as reducing and capping agent. Various parameters such as pH of gold solution, SL concentration and temperature were optimized for synthesis. Finally 400 µg/ml concentration of SL and 80 °C were found to be best for sophorolipid capped gold nanoparticles (AuNPs-SL) synthesis. UV-visible spectrum of the synthesized nanoparticles was obtained by measuring the absorption in the range of 300–700 nm with maxima at 530–540 nm (Fig. 1A). Measurement of size and potential was done using Malvern Zetasizer that suggested the average size of AuNPs-SL was 40 ± 10 nm with surface charge (ζ) −30 ± 3 mV (Shown in Supplementary Fig. S1 and Supplementary Table TS1). The zeta potential of AuNPs-SL is similar to the potential value of SL suggesting the capping of SL to the nanoparticles. Further, TEM studies were performed to know the shape and size of nanoparticles. The presence of faint white layer (after phosphotungstic acid, PTA staining) surrounding the nanoparticles (Fig. 1B-i) certified the capping of AuNPs by SL. TEM studies also revealed the presence of some spherical and clumped nanoparticles that indicates either incomplete separation of individual nanoparticles or nucleation state of nanoparticles synthesis that was also reflected in its size distribution graph done through DLS method using Malvern Zetasizer (Supplementary Fig. S1). As some of these nanoparticles were already clumped and precipitated upon storage (7–10 days) suggesting agglomeration over a period of time. To avoid formation of aggregated nanoparticles, modification in synthesis protocol was done by adding sodium borohydride (NaBH_4,_ represented as SBH). For this few drops of 100 mM sodium borohydride was used and thus, highly monodispersed sophorolipid capped gold nanoparticles reduced with NaBH_4_ (AuNPs-SL-SBH) were obtained.

The UV-Visible spectra of AuNPs-SL-SBH (Fig. 1A) have shown sharp absorption as compared to the AuNPs-SL indicating the uniform size distributed nanoparticles that was also reflected in size distribution graph and polydistribution index value (PDI, provided in Supplementary Fig. S1 and Table TS1) measured through Zetasizer and TEM (Fig. 1B-ii) micrograph. A lower PDI is related to the monodispersed colloidal solution. The zeta measurement value was similar to the earlier one (Supplementary Fig. S1 C,D). For further studies nanoparticles synthesized through subsequent method was used and indicated as AuNPs-SL only.

Capping by SL to AuNPs was also confirmed by thermogravimetric analysis (TGA). Figure 1C indicates two stage degradation of AuNPs-SL indicating either bilayering of AuNPs by SL or monolayering with some free SL suspended in solution. TGA graph represents nearly 40% mass loss that includes water molecules and the organic compound (sophorolipid). The mass loss up to 200 °C is mainly attributed by free water and strongly bound water^29^ (nearly 10% mass loss) and afterward due to sophorolipid^30^ (more than 20%). A similar pattern of thermal degradation was also observed by Niki *et al*.,(2013) where they found the multilayer decomposition of sophorolipids-functionalized iron oxide nanoparticles^31^. This data also suggest the compact packing of gold nanoparticles by sophorolipids.

Functional characterization of AuNPs-SL nanoparticles using FTIR indicated that the vibration peaks AuNPs-SL nanoparticles is almost similar to the sophorolipids [Fig. 1D(i,ii)]. The following important vibrational peaks were of SL aliphatic backbone (n = 2929–2926 & 2866–2856 cm^−1^), C = O group in carboxylic acid COOH (n = 1747–1738 cm^−1^), COH and CO of sophorose moiety (n = 1069, 1024 cm^−1^) confirming the capping of AuNPs-SL by sophorolipids. All these details affirm the complexation of sophorolipids to gold nanoparticles and further matches with earlier findings^31,32^. Thus, capping of SL to AuNPs was confirmed by FTIR and TGA.

### Antimicrobial activity

#### Agar well diffusion method

The AuNPs-SL was examined against Gram positive *S. aureus* and Gram negative *V. cholerae* by agar well diffusion method for their antibacterial activity. In this method, a particular volume of inoculum was spread over the agar plate. In which, the required number of holes were punched using borer for addition of antimicrobial compound and then plates were incubated for certain time interval. As the antimicrobial compound diffuses into the agar, growth inhibition of microbe is observed^33^. In this study, a clear zone of bacterial growth inhibition was observed in wells containing AuNPs-SL for both microbes after completion of incubation (Table 1). However, SL shows zone of inhibition (15 mm diameter) against gram positive bacteria *S. aureus* but not for Gram negative *V. cholerae*. Interestingly, AuNPs-Sigma did not exhibit any inhibition with either of microbes.

**Table 1:** The antimicrobial activity of AuNPs-SL by agar diffusion method against gram positive *Staphylococcus aureus* and gram negative *Vibrio cholerae*.

Samples	Zone of inhibition (in mm)	
	*S. aureus*	*V. cholerae*
AuNPs from Sigma	0	0
AuNPs-SL	15	15
SL	15	0
Ampicillin (50 µg/ml)	28	8

#### XTT assay

**Figure 2:**
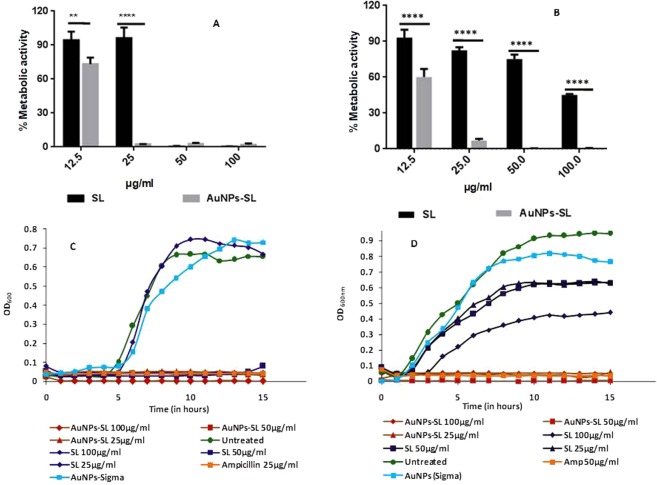
Antimicrobial activity of AuNPs-SL by XTT Assay and effect on growth kinetics against *S. aureus* (**A**,**C**) & *V. cholerae* (**B**,**D**). Bar graph (**A**,**B**) was analyzed using Two-way ANOVA, *P value < 0.01.

Antibacterial activity of the AuNPs-SL was further assayed against these microbes by serial double dilution method to determine the minimal inhibitory concentration (MIC) and viability by XTT [2, 3-bis(2-methoxy-4-nitro-5-sulfophenyl)-2H-tetrazolium-5-carboxanilide sodium salt] assay. McCluskey *et al*., (2005) described the MIC in the XTT assay as the lowest concentration of antimicrobial compound that prevents the colour change^34^. For *S. aureus* (Fig. 2A), MIC value was found to be either equal or twofold lower for SL (25–50 µg/ml) than AuNPs-SL (25 µg/ml). Contrary to this, *V. cholerae* remains viable even at 200 µg/ml of SL (the highest concentration used in assay). Interestingly, MIC of AuNPs-SL against *V. cholerae* (Fig. 2B) was 25 µg/ml and that is highly significant in comparison to sophorolipids. Interestingly, AuNPs-SL has shown higher efficacy against both the microbes with especial potency against Gram negative *V. cholerae*. Therefore, the efficiency of AuNPs-SL was further assessed against other Gram negative bacteria, *E. coli* that showed MIC at 12.5 µg/ml (Supplementary Fig. S2).

#### Growth kinetics

To further test cell viability in time-dependent manner, growth kinetics was also performed against *S. aureus* and *V. cholerae* at a different concentration (25, 50 and 100 µg/ml) for 15 h. Similar to XTT assay, the growth curve analysis also showed that sophorolipid has either equal or twofold lower activity than gold nanoparticles (Fig. 2C). At the highest concentration of SL (100 µg/ml) used against *V. Cholerae* in growth kinetics study, there is extended lag phase was observed whereas nanoparticles AuNPs-SL inhibited its growth even at 25 µg/ml (Fig. 2D). AuNPs-Sigma had no effect on growth of either of the microbes and has a similar trend of growth as of untreated cells.

Since AuNPs-SL has shown higher activity against *V. Cholerae* as comparison to SL. Therefore time dependant kill assay was performed only for *V. Cholerae*. We observed initial drop of cell density as comparison to untreated one that further recovered after certain time span (Supplementary Fig. S3). This observation suggested that SL imposes some stress due to its surfactant property to these cells that are recovered soon.

### Antibiofilm activity

#### Effect on biofilm formation

**Figure 3:**
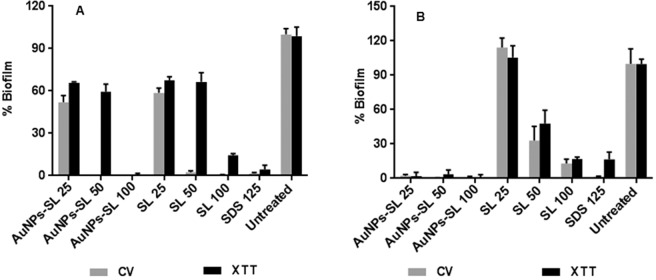
Antibiofilm activity of AuNPs-SL against *S. aureus* (**A**) and *V. cholerae* (**B**) by CV (grey colour) and XTT assay (black colour). Experiment was performed in triplicate and graph was plotted using GraphPad Prism 6.0 using average value with SD.

Biofilms have been defined as ‘aggregates of microorganisms in which cells are frequently embedded in a self-produced matrix of extracellular polymeric substances (EPS) that are adherent to each other and/or to a surface^35^. Biofilm is one of the survival strategies, in which the cellular metabolic activity gets reduced thus provides protection from different exogenic compounds etc. In general cells in the biofilm are more resistant to drugs^36^. Extracellular matrix is generally measured by crystal violet staining protocol; however, the technique has its own limitation as it cannot measure viable cells residing within the matrix of biofilm. Therefore, XTT was employed to measure the viability of cells residing within the matrix. The impact of biosynthesized AuNPs-SL was tested for inhibition potential against the mentioned microbes under given experimental conditions. Different concentration of AuNPs-SL and SL was used to check the antibiofilm activity against these microbes. SDS was used as positive control. The values were compared to negative control (without any treatment) and OD_590_ was plotted in terms of percentage (%). In case of *S. aureus*, (Fig. 3A) nearly 90% of biofilm formation was reduced at AuNPs-SL-50 and complete inhibition was found at the concentration AuNPs-SL 100 µg/ml. However upon performing antibiofilm assay through XTT method, complete inhibition of biofilm formation occurred at 100 µg/ml of AuNPs-SL while at 50 µg/ml AuNPs-SL, 60% remained as compare to the untreated. For SL also, similar pattern of inhibition was observed (Fig. 3A).

Interestingly, in case of *V. cholerae*, similar pattern of result was obtained by CV and XTT assay (Fig. 3B). Very low concentration i.e., 25 µg/ml of AuNPs-SL was enough to inhibit biofilm formation completely whereas SL inhibits the nearly 80% inhibition of biofilm at 100 µg/ml. This further confirms the effectiveness of AuNPs-SL against Gram negative bacteria.

#### Effect on mature biofilm

**Figure 4:**
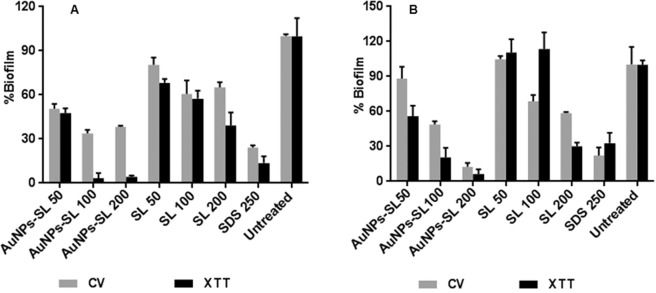
AuNPs-SL activity on preformed biofilm against *S. aureus* (**A**) and *V. cholerae* (**B**) by CV (grey colour) and XTT assay(black colour). Experiment was performed in triplicate and graph was plotted using GraphPad Prism 6.0 using average value with SD.

In the biofilm, cells are almost 1000 times or more than that resistant to planktonic cells that may differ from organism to organism. Various factors are responsible for this resistance such as growth rate, temperature, pH, nutritional status, etc. Another mechanism that is considered to be important is the slower diffusion of drugs. Hence, we checked the impact of AuNPs-SL activity on preformed biofilm of indicator microbes. For preformed biofilm, AuNPs-SL is found to be more effective than SL. The complete eradication of preformed biofilm of *S. aureus* (Fig. 4A) and *V. cholerae* ((Fig. 4B) occurred at the concentration of 100 µg/ml and, 200 µg/ml respectively. It is highly effective as compared to the positive control as shown in Fig. 4. Biosorption might be one of the reasons for inactivation of biofilm formation in case of silver nanoparticles mediated biofilm inactivation in *P. aeruginosa* as suggested by Park *et al*.^37^. The data in the present study validate that AuNPs-SL can effectively and rapidly detach biofilm, produced by *V. cholerae* and *S. aureus*, thus implies the application of the AuNPs-SL as biofilm-disrupting agents.

### Effect on non-dividing cells

**Figure 5:**
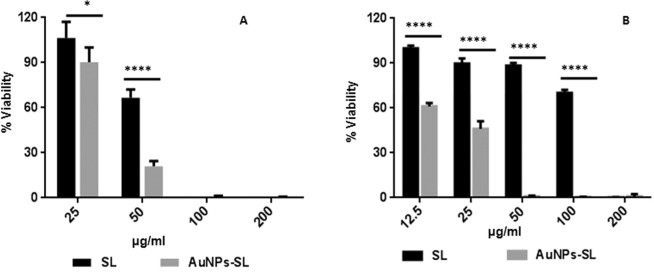
Antimicrobial activity on non-dividing cells of *S. aureus* (**A**) and *V. cholerae* (**B**) by XTT assay. Data was analyzed using Two-way ANOVA. *P value < 0.01.

Another survival strategy of microbes under adverse condition is dormant stage or non-multiplying stage. *V. cholerae* and *S. aureus* were used as indicator strains to produce long- duration stationary phase cells of Gram-negative and Gram-positive bacteria respectively. AuNPs-SL was found to kill non multiplying cells of both of these microbes (Fig. 5). On comparing its antimicrobial activity with SL, it was observed that AuNPs-SL was effective in killing in non-multiplying cells of *S. aureus* and *V. cholerae* (Fig. 5A,B respectively). In the case of *S. aureus*, nearly 80% inhibition was obtained at 50 μg/ml of AuNPs-SL respectively whereas less than 40% of inhibition led by SL at this concentration. Surprisingly, nearly 60% growth is reduced by AuNPs-SL (25 μg/ml) and completely inhibited at 50 μg/ml in *V. cholerae* whereas fourfold higher concentration of SL is needed to inhibit the non-dividing state cells.

### Evaluation of mechanism of action

#### SEM and TEM Imaging

**Figure 6:**
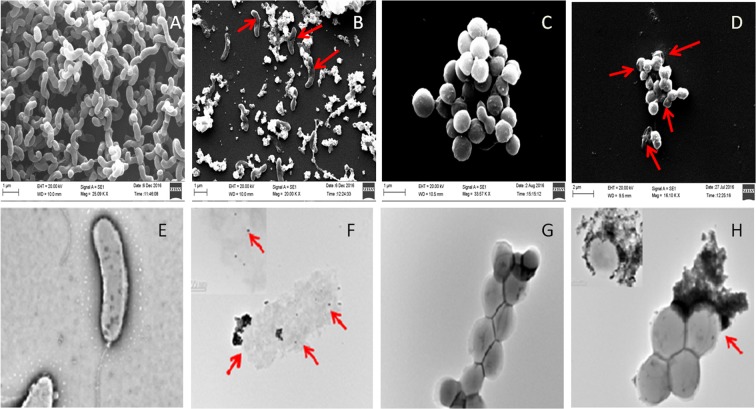
Untreated cells of *V. cholerae* (**A**,**E**) *S. aureus* (**C**,**G**) whereas AuNPs-SL treated cells *V. cholerae* (**B**,**F**) and *S. aureus* (**D**,**H**) by SEM (upper panel) and TEM (lower panel).

The cellular effect of AuNPs-SL against these microbes was visualized by SEM (Fig. 6A–D) and TEM (Fig. 6F–H). The untreated bacterial cells retained their original shape and demonstrated very smooth morphology (Fig. 6A,C). On the other hand, AuNPs-SL treated cells showed significant changes in morphology and cavities on the cells membranes (Fig. 6B,D). Upon treatment with the nanoparticles, the cells become irregular in shape and size observed in SEM and TEM images. Moreover, the figure also indicated the initiation of cell lysis and disruption of the cell membranes thus suggesting that the attachment of AuNPs-SL on the bacterial membrane might have resulted in cell rupturing. Internalization of nanoparticles to the cells was also observed in TEM micrograph for both pathogens (Fig. 6F,H)

#### Effect of AuNPs-SL on respiratory chain LDH activity in bacterial cells

**Figure 7:**
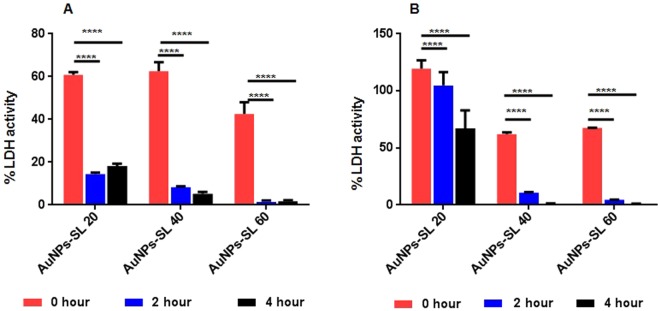
LDH activity of *S. aureus* (**A**) and *V. cholerae* (**B**). Average of triplicates was plotted with SD and data was analyzed using Two-way ANOVA (GraphPad Prism 6.0). *P value < 0.01.

Upon internalization of sophorolipid capped gold nanoparticles to the microbial cell can interfere with different components present inside the cells. One of the major components to which it can bind and interfere, is enzyme. The crucial enzyme system required for survival is electron transport system (ATP synthesis). Here, AuNPs-SL induced interference in cell respiration was measured through LDH activity (Fig. 7). In viable cells, the LDH activity was determined by measuring the reduction of NAD^+^ to NADH and H^+^ during the oxidation of lactate to pyruvate. In the second step of the reaction, diaphorase uses NADH and H^+^ to catalyze the reduction of a tetrazolium salt to a highly colored product, formazan. It was found that most of the cellular inhibition occurred at 2 hours of incubation at higher concentration i.e., 60 μg/ml; whereas lower concentration has partial inhibition of cellular activity. This result also suggested that the inhibition of respiratory enzymes in concentration and time dependent manner. Thus, it is clearly demonstrated that the activities of respiratory chain dehydrogenases in both *S. aureus* (Fig. 7A) and *V. cholerae* (Fig. 7B) were inhibited by AuNPs-SL, similar to the mechanism of action proposed by several groups^38^.

### Evaluation of synergistic effects between AuNPs-SL and antibiotics by broth microdilution checkerboard method

Checkerboard microdilution method was used to evaluate the synergistic effects of AuNPs-SL with three conventional antibiotics having different mechanism of action against these bacteria and the effects were evaluated by determining the FICI. The results of the synergistic effect are presented in Tables 2 and 3. An enhanced antibacterial synergistic activity of AuNPs-SL and two antibiotics (Kanamycin and polymyxin) was found against *S. aureus*, whereas with Amp it has shown additive effect. In the case of *V. cholerae*, AuNPs-SL has shown synergy with polymyxin, partial synergy with kanamycin and indifferent with ampicillin. The synergistic activities of AuNPs-SL in the presence of conventional antibiotics suggest that it might be possible to reduce the viability of bacterial strains at lower antibiotic concentrations. Most studies have been done on silver nanoparticles and similar types of interaction have also been reported by others^39^. This study demonstrates the synergistic effect of antibiotics and nanoparticles in improving their bactericidal property; it was suggested that nanoparticles can be effectively used in combination with antibiotics in order to improve their efficacy against various pathogenic microbes.

**Table 2:** MIC of individual drugs ampicillin, kanamycin, polymyxin and AuNPs-SL and FIC of these drugs with AuNPs-SL.

Species	Ampicillin	Kanamycin	Polymyxin	AuNPs-SL	MIC (µg ml^−1^)					
					Ampicillin + AuNPs-SL		Kanamycin + AuNPs-SL		Polymyxin + AuNPs-SL	
					FIC(A)	FIC(B)	FIC(A)	FIC(B)	FIC(A)	FIC(B)
*Staphylococcus aureus*	0.39	25	3125	50	0.195	25	6.25	6.25	390.65	12.5
*Vibrio cholerae*	16	32	3125	50	50	16	16	12.5	390.65	6.25

**Table 3:** FICI of drugs ampicillin, kanamycin, polymyxin with AuNPs-SL.

Species	Ampicillin + AuNPs-SL	FICI	Polymyxin + AuNPs-SL
		Kanamycin + AuNPs-SL	
*Staphylococcus aureus*	1(A)	0.375(S)	0.375(S)
*Vibrio cholerae*	2(I)	0.75(PS)	0.25(S)

FIC of antibacterial A = MIC of antibacterial A in combination/MIC of antibacterial A alone.

FIC of antibacterialB = MIC of antibacterial B in combination/MIC of antibacterial B alone.

FIC index (FICI) = FIC of antibacterial A + FIC of antibacterial B.

FICI < 0.5 synergy(S), 0.5 ≤ FICI < 1 partial synergy(PS),

FICI = 1 additive(A), 2 ≤ FICI < 4 indifferent(I), and 4 < FICI antagonism.

## Discussion

Synthesis of nanoparticles using chemical and physical methods is potentially unfriendly to the human health and environment. Here, sophorolipid; a glycolipid was used to greener synthesis of gold nanoparticles. Glycolipid mediated metal nanoparticles (silver nanoparticles) synthesis was first reported by Singh *et al*., (2009) and they have shown its antimicrobial activity^40^. Although silver nanoparticles and silver itself are well reported for antimicrobial activity but its cytotoxicity limits its application to human cell lines^41^. However gold nanoparticles are reported to least toxic nanoparticles among other metallic nanoparticles. Considering these points sophorolipid capped gold nanoparticles were synthesized in single step procedure that imposes antimicrobial property. This biosynthesis method for synthesizing AuNPs-SL has a distinct advantage over chemical synthetic techniques such as high efficiency, biocompatibility, ecofriendly and low toxicity to the environment. As per the available knowledge, we are the first to report the formation of gold nanoparticles synthesized directly from sophorolipid. UV visible absorbance spectra in the range of 530–540 nm confirmed the surface plasmon resonance of biosynthesized gold nanoparticles i.e., AuNPs-SL. TGA and FTIR provided additional strong evidence of biogenic synthesis and capping of SL to gold nanoparticles. Biosynthesized AuNPs-SL was examined for antimicrobial potential against different phase of life cycle achieved by two pathogenic strains Gram positive bacteria *S. aureus* and Gram negative bacteria *V. cholerae*. Results suggested AuNPs-SL has pronounced antimicrobial potential against different important pathogenic microorganisms with especial efficacy against Gram negative bacteria. Previous antimicrobial studies by various groups on the activity of SLs suggested better activity against Gram positive bacteria as compared to Gram negative bacteria^42,43^. Similar to earlier finding, in case of *V. cholerae*, growth inhibition was not observed at the experimental concentration of SL. This difference might be due to their cell wall and cell membrane composition^44^. It is considered that the antibiotic agents are freely diffused through the cell wall of Gram positive bacteria. However, the permeability of the outer membrane determines the diffusion of a given antibiotic agent in Gram-negative bacteria. This outer membrane is composed of proteins (porins) and an asymmetric lipid bilayer, in which outer and inner layer is mainly composed of lipopolysaccharides and phospholipids respectively. In this way, the membrane structure allows diffusion into the periplasmic space mediated through different pathways. These porins are involved in the efflux of various compounds such as different groups of antibiotics. The alteration in porin composition in order to reduce the influx of compounds is also major threat towards antibiotic resistance imposed by Gram negative bacteria. These reasons might have led to differences in the zone of inhibition and MIC in these two pathogens upon treatment with AuNPs-SL and SL.

Under environmental stress conditions, bacteria respond differently such as changing the morphology, changing cellular composition, forming biofilm and attaining non multiplying state. Biofilm and non multiplying phase of bacteria are imposing a major health problem in different aspects such as prosthetic valves, catheters and contact lenses. Since most of available drugs are based on the metabolically active cells therefore they fail to act against non-multiplying bacteria that ultimately leads to slow or partial loss of infected tissue. Apart from this repeated administration of drugs or antibiotics fails to act effectively. Here, results have shown the AuNPs-SL as promising bactericidal agent for non dividing cells too.

With the use and misuse of antimicrobials, most of the pathogens develop resistance to multiple antibiotics thus reducing the effectiveness of drugs. Multidrug resistance (MDR) have emerged as major threats public health. Repurposing of drugs and combinatorial therapy are the main approaches that are adopted to combat this issue this issue. Metal nanoparticles have also shown new aspects to address this problem. Combinatorial therapy including the gold nanoparticles (here AuNPs-SL) with polymyxin and kanamycin have shown synergy against both pathogenic microbes. Polymyxin B binds to the cell membrane and alters its structure and makes it more permeable. AuNPs-SL exhibits synergy with polymyxin indicating the similar kind of bactericidal activity performed by these nanoparticles. Along with this, Kanamycin that affects the protein synthesis machinery, also exhibits synergy with AuNPs-SL. It reduces drug doses by eight fold in case of polymyxin for both microbes whereas fourfold and twofold for kanamycin in Gram positive and Gram negative respectively. In this way, it reduces the drug burden imposed by a single drug, hence improve the clinical outcomes. Since gold nanoparticles posses least cytotoxicity among other metal nanoparticles thus it is safe to use for medical applications.

However the exact mechanism of antimicrobial activity exhibited by metal nanoparticles is unclear. But, synergistic effects shown by AuNPs-SL with polymyxin and kanamycin provide an idea towards the mechanism of its action. SL is known to be effective against the Gram positive bacteria but least for Gram negative bacteria. The uncapped gold nanoparticles do not exhibit antibacterial activity against the both of these microbes. However, capping of SLs to the gold nanoparticles mediates the antibacterial activity not only to the Gram positive but also Gram negative bacteria with higher efficacy for later one. SL mediates the formation of pores and disruption of cell membrane due to its surfactant like properties and also helps in the internalization of AuNPs-SL, which was also observed in TEM images. As evidenced by LDH assay, AuNPs-SL interferes with the metabolism upon internalization by binding to the enzymes and blocking its activity. Available reports (silver nanoparticles) of mechanism for antimicrobial activity suggests the binding ability with the cell wall and altering the cell membrane permeability, accumulation inside the cells and interaction to the respiratory enzyme are the major reasons or killing effect of microbes. We also observed a similar kind of findings as reported earlier. Thus, it can be said that AuNPs-SL exhibits its antibacterial properties by binding to cell membrane, disrupting cell membrane integrity, leakage of intracellular materials and interfering the enzyme activity.

As the emergence of resistance imposed by Gram negative bacteria is more critical and hard to treat with the limited number of antimicrobial agents thus major concern to deal with this issue. AuNPs-SL is found to be very promising to handle this concern. AuNPs-SL can cross the barrier of biofilm and kills the residing cells effectively. Due to this property, it can prevent the emergence of resistance cells or persister cells. Thus this study provides the idea of using AuNPs-SL as antibacterial agents that have three major significant over existing antibiotic therapy (i) active against Gram positive and Gram negative bacteria both, (ii) have synergy with different antibiotics thus reducing drug burden, minimise side effects and (iii) disrupting the biofilm and non dividing cells thus minimising the existence of persisters. Altogether it can be said that sophorolipid capped gold nanoparticles can be a promising agent for antimicrobial therapy (especially for Gram negative bacteria) having different mode of bactericidal action.

## Experimental section

### Materials and methods

Gold (III) chloride hydrate (50790), Gold nanoparticles (Uncapped and dissolved in PBS, catalogue no. 752584), ampicillin (A 9518) and kanamycin (60615), XTT (X4626) were purchased from Sigma-Aldrich. Luria-Bertini (LB) medium, yeast extract, peptone, dextrose and agar used for culture growth were purchased from Himedia.

### SL production

*Stamerella bombicola* (MTCC 1910) was used for SL production, and it was maintained on YPD plate [yeast extract (10 g/L), peptone (10 g/L) and dextrose (20 g/L) and 2% agar]. For inoculum preparation, *S. bombicola* was grown in YPD broth for 2 days at 30 °C with agitation (200 rpm) in orbital shaker. From this preparatory inoculum 2% inoculum was added to production medium and cultured for 7 days at 200 rpm at 30 °C as suggested by Faraz *et al*.^45^ provided in supplement.

### Synthesis of AuNPs-SL

#### Synthesis and characterization

Sophorolipid mediated gold nanoparticles (AuNPs-SL) were synthesized by two methods. In the first method, 40 µl of sophorolipid (100 mg/ml) was added to 10 ml of chloroauric solution (400 µg/ml, pH 5.5 ± 0.2) mixed properly and kept at 80 °C till the appearance of characteristic red colour. Since this method took several hours of synthesis, therefore first method was modified by adding few drops of sodium borohydride (NaBH_4,_ 100 mM) during the gold solution preparation.

Initial characterization of SL capped gold nanoparticles (AuNPs-SL) was done using dual beam UV visible spectroscopy (Hitachi U-2900) by measuring the absorption in the range of 300–700 nm. Measurement of size was carried out using dynamic light scattering (DLS) method and surface potential was measured using Malvern Zetasizer. Further, morphological and topological analysis was performed using TEM (JEOL 2100) imaging for which sample was stained with 2% phosphotungstic acid (PTA). Fourier transform infrared (FTIR, Perkin Elmer, Model: Spectrum 100) spectra of SL and AuNPs-SL was recorded five times per scan in the frequency region 4000–400 cm^−1^ at room temperature by KBr pellet method. Thermo gravimetric analysis (TGA) of purified powders of SL and dried AuNPs-SL was performed on a TGA-7 Perkin-Elmer instrument at a scan rate of 10 °C min^−1^.

### Bacterial strain and culture conditions

Two pathogenic strains, Gram-positive *Staphylococcus aureus* MTCC 1430 and Gram-negative *V. Cholerae* EL Tor strain N16961 were used as indicator organism for antimicrobial activity in this study. Bacterial strains were revived on LB agar (2%) plate media from glycerol stock (15%). For broth culture, strains were grown in LB broth from LB agar plate and kept at 37 °C, 200 rpm for overnight and then subcultured for 2–3 hours to obtain log phase cells^46–48^. Generally, cultures were diluted in ratio of 1:1000 in LB broth, from which 100 μL of culture was either spreaded on agar plate or added in microtiter plate for some assay else mention in the particular section.

### Antimicrobial activity

#### Agar well diffusion method

Initially, the antibacterial activity of AuNPs-SL and SL was performed against indicated bacterial strains by agar diffusion method^49^. 10^7^ CFU/ml of cells was spread on LB agar media separately in three replicate plates. 6 mm diameter of wells was made on these agar plates using agar well borer and 100 μl of the following samples; AuNPs-SL (400 μg/ml), SL (400 μg/ml), Ampicillin (50 μg/ml) and AuNPs-Sigma were loaded separately into the wells. Plates were kept in incubator at 37 °C for overnight. The antimicrobial activity was calculated by measuring the diameter of zone of bacterial growth clearance on agar plate. Here, it is worth to write that all the concentration of gold nanoparticles mentioned here are in respect of SL concentration.

#### XTT assay

The AuNPs-SL and SL were taken in the range of 200–6.25 µg/ml with serially two fold diluted in 96 well plates. The concentration mentioned here is in terms of SL concentration. For controls, ampicillin (20 µg/ml), AuNPs from Sigma and media blank were taken. 100 µl of the log phase culture (10^7^ CFU/ml) was added to the plates, mixed well with testing compounds and incubated for 16 hours at 37 °C. Later on, bactericidal effect of AuNPs-SL and sophorolipid was investigated by colorimetric XTT reduction assay. The assay is based on the conversion of tetrazolium salt XTT to form water-soluble coloured formazan product by metabolic active cells that can be measured spectrophotometer^50^. XTT solution (0.5 mg/ml) was prepared in 1X PBS and menadione (1 mM) in acetone respectively. Prior to each assay, these solutions were thawed and mixed to give final concentration of 1 μM of menadione. From this, 30 μL of XTT was added to each well of microplate and incubated at 37 °C in dark for 2 hours. The change in colour due to viability of cells was measured using microplate reader (Biotek Spectrophotometer, Power wave XS2) at 490 nm. Experiments were performed in triplicate and any inferences of nanoparticles in the measurement have been deducted from the absorbance imposed by samples and then average value is reported with ±SD.

#### Growth kinetics

Growth kinetics studies of both the organism were performed in honey comb 100 well plate using Bioscreener reader. 100 µl of the log phase culture (containing 10^7^ CFU/ml of cells) was seeded with different concentration of the AuNPs-SL & SL (100, 50 and 25 μg/ml) in 100 well honey comb plate. Negative (without any treatment) and positive controls (ampicillin) were taken properly as shown in the graph 2C and D. Uncapped AuNPs (AuNPs-Sigma) was also used for the study. The plate was kept at 37 °C with continuous shaking in the Bioscreener reader for 16 h and OD_600_ was measured at equal interval of 1 h. The experiment was done in triplicates for each sample set, average was calculated and graph was plotted.

### Antibiofilm activity

Effect of AuNPs-SL on inhibition of biofilm formation and mature biofilm eradication was studied in 96 well microtiter plates by two methods; Crystal Violet assay and XTT assay. To study the extent of biofilm formation, the strains *S. aureus* and *V. cholerae* were grown overnight in LB broth media at 37 °C in orbital rotatary shaker that was further diluted to 1:100 into fresh LB sucrose (1% sucrose)^48^ and LB saline (2% NaCl)^47^ media respectively. *V. cholerae* is a halophilic microbe and under stress condition, biofilm formation is induced^51^. 100 μl of the diluted culture was put into each well of the 96 well plates containing different concentration of AuNPs-SL and SL (25, 50 and 100 μg/ml) that was incubated for 24 h at 37 °C. SDS (125 μg/ml) treated and untreated cells of these pathogens were taken as control. Planktonic cells were removed after completion of incubation time and washed thrice with PBS (pH 7.2). The extent of biofilm formation was measured by CV and XTT assay as mentioned below. All the experiments were done in triplicate and the average value was expressed in extent of % biofilm that was normalized with untreated cells.

#### Eradication of preformed biofilm

In case of mature (preformed) biofilm eradication study, the strains *S. aureus* and *V. cholerae* were grown overnight at 37 °C in orbital rotatary shaker that was further diluted to 1:100 into fresh LB sucrose (1% sucrose) and LB saline (2% NaCl) media respectively. 100 μl of the diluted culture was put into each well of the 96 well plates without any treatment to form biofilm. Planktonic cells were removed, aspired thrice with PBS, then treated with AuNPs-SL and SL (50, 100 and 200 μg/ml) and incubated for another 4 h at 37 °C. Cells with SDS and without AuNPs-SL served as the positive and negative controls respectively. All the experiments were done in triplicate and the mean value was expressed in extent of biofilm in terms of percentage that was normalized with untreated cells.

$$ \% {\rm{Biofilm}}=({\rm{Treated}}/{\rm{Untreated}})\ast 100$$

#### Measurement of biofilm inhibition

*Crystal violet (CV) staining method*: This was carried out as described by George A. O’Toole (2011) with slight modification^52^. Briefly, biofilm cells remaining were fixed using methanol for 15 minutes and dried at room temperature. Biofilm was stained with 0.1% CV for 15 minutes followed by washing with PBS to remove excess stain and stained biofilm was dried properly. Finally, suspension of the biofilm was done in 30% acetic acid followed by quantification by measuring the absorbance at 595 nm. The values obtained were considered as an index of bacteria adhering to the surface of well wall and extra cellular mass produced by them.

$$ \% {\rm{Biofilm}}=({{\rm{OD}}}_{595}\,{\rm{of}}\,{\rm{treated}}\,{{\rm{cells}}/{\rm{OD}}}_{595}\,{\rm{of}}\,{\rm{untreated}}\,{\rm{cells}})\ast 100$$

*XTT method*: For XTT assay, aspired cells were resuspended into 1% glucose containing XTT-menadione solution and incubated for two hours at 37 °C in dark. Then measurement was taken at 490 nm in ELISA plate reader. Here the OD_490_ is reflection of viable cells residing within the biofilm.

$$ \% {\rm{Biofilm}}=({{\rm{OD}}}_{490}\,{\rm{of}}\,{\rm{treated}}\,{{\rm{cells}}/{\rm{OD}}}_{490}\,{\rm{of}}\,{\rm{untreated}}\,{\rm{cells}})\ast 100$$

### Effect on non-dividing cells

#### Preparation of non-dividing cells

Non-dividing state of *S. aureus* cells was obtained following the method described by Hu *et al*.^53^. The culture was grown in LB medium at 37 °C, 200 rpm for seven days. Cells were centrifuged and washed twice with PBS (pH 7.2), resuspended in the same buffer and further incubated at 37 °C and 200 rpm for 7 days. Cell viability was checked by counting colony-forming units (CFUs) every 24 h. The culture showed a decrease in CFU/ml in the first 5 days, and remained constant thereafter, at 4 × 10^5^. From this, 100 μl of cells were seeded into microtiter plate for study.

Non-dividing state of *V. cholerae* was obtained using the method as described earlier^54,55^. *V. cholerae* was inoculated into alkaline peptone water for 16 hours at 37 °C in shaker then cells were collected by centrifugation, washed thrice with 1% artificial seawater and resuspended. The final concentration of cells was maintained as 10^8^ CFU/ml in one litre flask containing 200 ml of 1% artificial sea water and incubated at 4 °C in the dark, without shaking. After one month, cell viability was performed by colony forming unit on LB agar plate for 3–5 consecutive days to know the constancy of viable cell numbers. Further, 100 μl of 4 × 10^6^ CFU/ml was used for the activity analysis in 96 well microtiter plate.

#### Antimicrobial activity assay against non-dividing cells

100 μl of non-multiplying cell suspension of both strains were mixed with equal volume of different concentration of AuNPs-SL and SL in concentration range of 25–200 μg/ml in separate microtiter plate and incubated for 16 h at 37 °C. An untreated sample, taken as control, was processed in the same way. Viability was checked by XTT assay and mean value was used for plotting graph viability.

$$ \% {\rm{Viability}}=({\rm{B}}/{\rm{A}})\ast 100$$

where; A = OD_490_ of untreated cells, B = OD_490_ of treated cells.

#### SEM and TEM imaging

The morphological changes induced by nanoparticles against these strains were visualized by using Scanning Electron Micrograph (SEM) and Transmission electron microscopy (TEM). For SEM analysis, untreated and nanoparticles treated cells were prepared following established protocol^56^. 10^7^ CFU/ml of cells in 5 ml of LB broth media was treated with AuNPs-SL-25 μg/ml and incubated for 2 hours in orbital shaker at 37 °C. Cells were then centrifuged and washed with fresh PBS thrice. In brief, treated and untreated cells were fixed on coverslip and incubated in 2% glutaraldehyde solution for half an hour. Washing with PBS pH 7.2 was followed by dehydration with 30%, 50%, 70%, 90% and 100% ethanol. Final dehydration was done with tertiary butyl alcohol and desiccated in lyophilizer. Samples were coated with gold sputter before imaging in SEM (Zeiss Evo 40).

For transmission electron microscope (TEM), samples were prepared by adding a few drops of treated and untreated cells on carbon coated grid and incubated for 10 minutes. Remaining samples were removed using blotting sheets and then stained using 2% phosphotungstic acid for 10 minutes. Extra stain was removed similarly and grid was dried for half an hour before imaging.

### Effect of AuNPs-SL on respiratory chain LDH activity in bacterial cells

To investigate the oxidative stress induced damage on respiratory system by nanoparticles, LDH activity was checked^57^. Overnight grown culture was subcultured to obtain log phase cells and diluted in such a way that 10 ml of media contains 10^8^ CFU/ml cells. Different concentration of AuNPs-SL and SL (0, 20, 40 and 60 µg/ml) was mixed to cultures and incubated for 4 hours. At different time intervals (2 hours), 1 ml of samples were withdrawn, centrifuged, washed thrice with PBS (pH = 7.2) and LDH activity was measured as per manufacturer instruction. The plate was then incubated with gentle shaking on an orbital shaker for 30 min at room temperature and reaction was stopped by adding stop buffer solution. After incubation, the OD_495_ was measured and treated samples were compared with untreated assigning untreated cells activity as 100%. All the experiments were performed in triplicate and data are expressed as means ± SD.

### Checker board assay

A checkerboard microdilution technique was used to explore the synergism between the antibiotics and AuNPs-SL against these microbes bacteria i.e., *S. aureus* and *V. cholerae*. In this assay, minimum inhibitory concentration (MIC) was determined, for antibiotics and AuNPs-SL individually and in their paired combinations^58^. The extent of synergy between antibacterial drugs is often expressed as fractional inhibitory concentration (FIC). Three antibiotics drugs, namely ampicillin (Amp), kanamycin (Kan) and polymyxin B sulphate (Poly) were used to investigate their synergy with prepared AuNPs-SL against these microbes. Bacterial cultures were grown as mentioned earlier and from this, 100 μl of was added to the same 96 well microtiter plate containing different dilution of drugs in combination of AuNPs-SL or separately drug and AuNPs-SL, incubated at 37 °C for 16 h in bioincubtor. Afterwards, XTT assay was performed to determine the MIC and FIC of antibiotics and AuNPs-SL. The lowest concentration at which no change in colour of XTT occurred was noted as the MIC value of the individual and combined test agents. FIC was evaluated from the MIC of antibacterial compound A and the MIC of antibacterial compound A in combination with test agent B. Therefore,

FIC of antibacterial A = MIC of antibacterial compound A in combination/MIC of antibacterial compound A alone.

In similar manner, FIC of antibacterial compound B was calculated and the ΣFIC index was done by adding these two FIC agents.

$${\rm{FIC}}\,{\rm{index}}={\rm{FIC}}\,{\rm{of}}\,{\rm{antibacterial}}\,{\rm{A}}+\,{\rm{FIC}}\,{\rm{of}}\,{\rm{antibacterial}}\,{\rm{B}}$$

The calculated FIC index was used as indicator of the nature of interaction between the two tested antimicrobial agents as suggested by American Society of Microbiology^59^.

### Statistical analysis

Statistically significant differences between the groups were determined using two-way ANOVA and multiple pairwise comparison procedures. Statistical analysis was performed using GraphPad Prism 6 software.

## Supplementary information

Supplementary information
