# Supplementary material for: Facile One Pot Greener Synthesis of Sophorolipid Capped Gold Nanoparticles and its Antimicrobial Activity having Special Efficacy Against Gram Negative Vibrio cholerae
Source: Sci Rep. 2020 Jan 29;10:1463. doi: 10.1038/s41598-019-57399-3 (PMC6989514; doi:10.1038/s41598-019-57399-3)
Supplement: Supplementary file 1 — Supplementary information [file 41598_2019_57399_MOESM1_ESM.docx]

**Facile One Pot Greener Synthesis of Sophorolipid Capped Gold Nanoparticles and its Antimicrobial Activity having Special Efficacy Against Gram Negative *Vibrio cholerae***

Sristy Shikha, Saumya Ray Chaudhuri, Mani Shankar Bhattacharyya*

CSIR-Institute of Microbial Technology (IMTECH), Sector-39A, Chandigarh 160036, India.

E mail: [sristyshikha@imtech.res.in](mailto:sristyshikha@imtech.res.in); [manisb@imtech.res.in](mailto:manisb@imtech.res.in)

Phone +911726665313; Fax: +91-172-2695215.

**Production and purification of Sophorolipid**

Sophorolipid (SL) production and purification was done as suggested by Faraz et. al., (2016).^1^ For the production of SL *Starmerella bombicola*, MTCC 1910, was used. The culture was grown in YPD broth for 2 days at 30°C with agitation (200 rpm) for inoculums preparation in orbital shaker. From preparatory media, 2% of inoculum was added to production medium and cultured at 30°C, 200 rpm for 7 days. Further cell mass were separated from the culture broth by centrifugation at 6000 rpm for 10 minutes and extracted using ethyl acetate in 1:1 ratio. The ethyl acetate extract was vacuum dried at 45°C and residual hydrophobic components were washed with n-hexanes to give crude SL mixtures. This crude mixture was further purified from different ratio of methanol and chloroform by passing through silica column (mesh size 60-120).

**Time dependent kill assay**

*V. cholerae* was subjected to time kill assay in dose dependent manner in presence of AuNPs-SL and SL.^2^ The strain was overnight grown in LB broth media from which secondary culture was inoculated. Bacterial inoculum equal to 2 x 10^7^ CFU/ml were added to LB broth (5 ml) containing different concentration of AuNPs-SL and SL (50 and 100 μg/ml) and the volume was adjusted to 10 ml by adding LB broth. The control was also run in parallel without any treatment. A 100 µl of culture broth was harvested at 0 h, 1h, 2 h, 4 h and 6 h followed by 10-fold serial dilutions of the inoculums in normal saline and plating on LB agar plates. After incubation at 37 °C for 16 h, the colonies were counted.

Figure S1: Size distribution histogram of AuNPs-SL in absence (A) and presence (B) of sodium borohydride by DLS method; Zeta measurement of AuNPs-SL in absence (A) and presence (B) of sodium borohydride using Melbern Zetasizer

Figure S2: Antimicrobial activity of AuNPs-SL by XTT Assay against *E. coli*

Figure S3: *In vitro* time and dose dependent bactericidal of effect AuNPs-SL and SL on *V. cholerae*.

Table TS1: Size, PDI and Zeta potential value of nanoparticles measured through Melbern Zetasizer

| Sample |  | Avg Size (diameter nm) | | PDI | Zeta Value (ζ) (in mV) |
| --- | --- | --- | --- | --- | --- |
| AuNPs-SL-SBH | | 40±10 | 0.389 | | 30±3 |
| AuNP-SL | | 30±5 | 0.281 | | 29±3 |

**References**

1. F. Haque, M. Alfatah, K. Ganesan and M. S. Bhattacharyya, *Scientific reports*, 2016, **6**.

2. R. R. Cutler, M. Odent, H. Hajj-Ahmad, S. Maharjan, N. J. Bennett, P. D. Josling, V. Ball, P. Hatton and M. Dall'Antonia, *Journal of Antimicrobial Chemotherapy*, 2008, **63**, 151-154.
